# Book Review: Underexplored Medicinal Plants From Sub-Saharan Africa: Plants With Therapeutic Potential for Human Health

**DOI:** 10.3389/fphar.2020.00965

**Published:** 2020-06-26

**Authors:** François Chassagne, Mark Morgan

**Affiliations:** ^1^ Center for the Study of Human Health, Emory University College of Arts and Sciences, Atlanta, GA, United States; ^2^ School of Natural Resources, University of Missouri, Columbia, MO, United States

**Keywords:** medicinal plants, herbal monograph, pharmacopeia, Africa, Mascarenes

Use of plants as traditional medicine plays a significant role in primary health care for many individuals in developing countries. The sale of herbal supplements and remedies to those who use plants as alternative medicine is responsible for a multibillion-dollar, nonpharmaceutical industry (Smith et al., 2019). To date, 28,187 plant species have been used for preventing or treating an array of health disorders. However, only 4,478 of them are mentioned in regulatory publications, indicating a need for more rigorous scientific information on safety and efficacy (Willis, 2017).

This book provides some valuable information on 46 understudied medicinal plant species in sub-Saharan Africa, including evidence-based descriptions of their use in medicine (Lall, 2020). Chapters actually consist of monographs that compile information on each of them, including references. Monographs are formatted in such a way that makes information easy to locate. For example, the template is organized into eight units: a general description, botanical description, distribution, ethnobotanical usage, phytochemical constituents, thin layer chromatography (TLC) fingerprinting, pharmacological properties, and additional information. The last section summarizes the scientific data and gives a quick overview of the medicinal potential for each plant, ideally suited for non-expert readers. Chapters also include photographs to represent different views of the plant, one thin-layer chromatogram used as a “fingerprint” to identify the plant chemistry, one map showing its geographical distribution throughout sub-Saharan Africa, and chemical structures of any known natural products found in the plant.

This book not only contributes to our scientific knowledge of medicinal plants, but also expands our understanding of African pharmacopeia. The 46 plant species described in this book grow in the sub-Saharan region and belong to the understudied medicinal plant species from this area. Only two plants described in this book are present in the current African Herbal Pharmacopeia (AHP), one of the most famous compilations of African medicinal plants. In the AHP, 51 medicinal plants are described. Therefore, this book makes a large contribution to African pharmacopeia by doubling the number of monographs in the AHP.

Namrita Lall, editor, is a scientist recognized all over the world for her work on medicinal plants. This confirms the high value of this book. Much of it has been written by scientists from the University of Pretoria in South Africa who have a long-standing tradition of research on medicinal plants. Chapter authors include renowned botanists, ethnobotanists, and phytochemists. A great contribution has been made by scientists who specialize in the flora of Mascarenes (Mauritius, Reunion, and Rodrigues islands) with about 13 species described. Thanks to the high degree of endemism on these islands, most of the plant species present are limited geographically to this archipelago. They represent a great source of phytomedicines since most of these species are poorly studied and could reveal the presence of some unique phytochemicals with original structure and significant pharmacological activity. For example, *Phyllanthus phillyreifolius* found in Mauritius and Reunion islands possess an interesting polyphenolic compound: geraniin. Aside from plant species located on the Mascarenes islands, all of the rest were found in mainland Africa, especially South Africa (except for *Newtonia buchananii*). Three species were endemic to South Africa and neighboring countries (i.e., *Greyia radlkoferi*, *Plantago longissima, Plectranthus neochilus*), and one was endemic to Madagascar (i.e., *Ravenala madagascariensis*).

The 46 species described in this book belong to 35 different genera and 27 different botanical families, representing a high amount of taxonomical diversity. With five species highlighted, *Aloe* is the most studied genus. Four of five *Aloe* species are proposed for treating wound healing or skin disorders. This is congruent with medicinal uses of the famous species from this same genus: *Aloe vera* which is widely known for its wound healing properties. Besides, it appears that isoorientin, a compound not detected in *A. vera* is responsible for the high antimicrobial properties found in *Aloe macra.* While some plant species cited in this book are close relatives to popular species from the same genus, others have a limited number of species. Such is the case of *Heteropyxis canescens* and *Heteropyxis dehniae* which belong to a genus that consists of three species.

One of the main strengths of this book lies in its potential for validating herbal medicines by compiling data on the safety and efficacy of selected plant species. Of particular interest is the species *Euclea natalensis* which exhibited good antimycobacterial activity *in vitro* and *in vivo*, and which was found to have low, if any, observable toxicity. As noted in the book, this plant species has great potential as a possible adjuvant for patients suffering from tuberculosis. Another plant species with safe and effective medicinal potential is *Commelina benghalensis* which might be developed as an antidiarrheal or anthelminthic agent. The book also provides some beneficial information regarding safe uses of plants. For example, *Zantedeschia aethiopica* must be boiled or cooked prior to consumption because raw plant material can cause swelling of the throat due to the presence of calcium oxalate crystals.

Our main criticism focuses on the last section of the monograph: “Additional information.” A simple reorganization could have provided some more useful recommendations for the public. For example, no data related to dose or posology is shown in the dosage subsection for any plant. Only “formulated cream” is found in this subsection and thus a more appropriate title could have been used: e.g., “method of administration and preparation,” and included some more information. Also, one subsection proposing the part(s) of plant to be used would have been helpful to complete the subsection “Therapeutic (proposed) usage.”

Overall, this book will be highly prized by readers from different backgrounds. Pharmacognosists and phytochemists will appreciate the description of compounds isolated and biological data provided in the book. Ethnobotanists will value the traditional uses detailed in each monograph. International and national agencies can use this book to guide their policy relative to quality control and identification of medicinal plants. Students from various fields, especially from pharmacognosy, will learn about standardized methods used to create monographs. Finally, amateur botanists and persons interested in understudied medicinal plants will be able to recognize them in the field using high-quality photographs found in this book. Overall, this book is a useful and accessible resource on selected plants from sub-Saharan Africa which contributes to their safe and effective use in traditional medicine. We recommend it to anyone who is interested in medicinal plants.

## Author Contributions

FC and MM wrote the manuscript and approved the final manuscript.

## Conflict of Interest

The authors declare that the research was conducted in the absence of any commercial or financial relationships that could be construed as a potential conflict of interest.
